# Imagining fictitious and future experiences: Evidence from developmental amnesia

**DOI:** 10.1016/j.neuropsychologia.2010.06.037

**Published:** 2010-09

**Authors:** Eleanor A. Maguire, Faraneh Vargha-Khadem, Demis Hassabis

**Affiliations:** a Wellcome Trust Centre for Neuroimaging, Institute of Neurology, University College London, 12 Queen Square, London WC1N 3BG, UK; b Developmental Cognitive Neuroscience Unit, Institute of Child Health, University College London, 30 Guilford Street, London WC1N 1EH, UK; c Gatsby Computational Neuroscience Unit, University College London, 17 Queen Square, London WC1N 3AR, UK

**Keywords:** Scene construction, Episodic memory, Future thinking, Hippocampus, Autobiographical, fMRI

## Abstract

Patients with bilateral hippocampal damage acquired in adulthood who are amnesic for past events have also been reported to be impaired at imagining fictitious and future experiences. One such patient, P01, however, was found to be unimpaired on these tasks despite dense amnesia and 50% volume loss in both hippocampi. P01 might be an atypical case, and in order to investigate this we identified another patient with a similar neuropsychological profile. Jon is a well-characterised patient with developmental amnesia and 50% volume loss in his hippocampi. Interestingly both Jon and P01 retain some recognition memory ability, and show activation of residual hippocampal tissue during fMRI. Jon's ability to construct fictitious and future scenarios was compared with the adult-acquired cases previously reported on this task and control participants. In contrast to the adult-acquired cases, but similar to P01, Jon was able to richly imagine both fictitious and future experiences in a comparable manner to control participants. Moreover, his constructions were spatially coherent. We speculate that the hippocampal activation during fMRI noted previously in P01 and Jon might indicate some residual hippocampal function which is sufficient to support their preserved ability to imagine fictitious and future scenarios.

## Research highlights

▶ Jon is a patient with developmental amnesia and bilateral hippocampal damage. ▶ Despite this, he is able to richly imagine fictitious and future scenarios. ▶ This is in contrast to most patients with adult-acquired amnesia. ▶ Residual hippocampal function may underpin preserved simulation ability.

## 1 Introduction

The hippocampus is part of a network of brain regions acknowledged to play a role in retrieving autobiographical memories (Cabeza & St Jacques, 2007; Maguire, 2001; Spreng, Mar, & Kim, 2009; Svoboda, McKinnon, & Levine, 2006) and supporting spatial navigation (Bird & Burgess, 2008; Burgess, Maguire, & O’Keefe, 2002). In the last few years, functional MRI (fMRI) findings have indicated the hippocampus is also involved in imagining fictitious episodes (Hassabis, Kumaran, & Maguire, 2007; Summerfield, Hassabis, & Maguire, 2009, 2010), and the simulation of plausible personal future events (e.g. Addis & Schacter, 2008; Addis, Pan, Vu, Laiser, & Schacter, 2009; Addis, Wong, & Schacter, 2007; Botzung, Denkova, & Manning, 2008; Okuda et al., 2003; Szpunar, Watson, & McDermott, 2007). Further compelling evidence for this comes from patients with damage thought to be relatively restricted to the hippocampus bilaterally. Hassabis, Kumaran, Vann, and Maguire (2007; see also Klein, Loftus, & Kihlstrom, 2002; Rosenbaum et al., 2005) tested five patients with such damage that was acquired in adulthood, rendering them amnesic. They were asked to imagine and describe fictitious scenarios and also possible plausible future episodes. The patient group was significantly impaired relative to control participants on both tasks, and a possible source for their deficit was identified. Whilst patients were able to produce relevant details when asked to imagine, their descriptions lacked spatial coherence and were fragmented. It was concluded that the hippocampus may play a critical role in imagination by binding together the disparate elements of an event or scene (Cohen & Eichenbaum, 1993; Hassabis, Kumaran, Vann, et al., 2007; O’Keefe & Nadel, 1978).

The involvement of the hippocampus (and other brain areas) in supporting apparently disparate functions such as autobiographical memory, spatial navigation, imagination, and future thinking, led Hassabis and Maguire (2007, 2009) to propose that they were underpinned by a common set of processes which they described as ‘scene construction’. This involves the mental generation and maintenance of a complex and coherent scene or event. This is achieved by the reactivation, retrieval and integration of relevant semantic, contextual and sensory components, stored in their modality specific cortical areas (Wheeler, Petersen, & Buckner, 2000), the product of which has a coherent spatial context (Hassabis, Kumaran, Vann, et al., 2007), and can then later be manipulated and visualised.

**Fig. 1:**
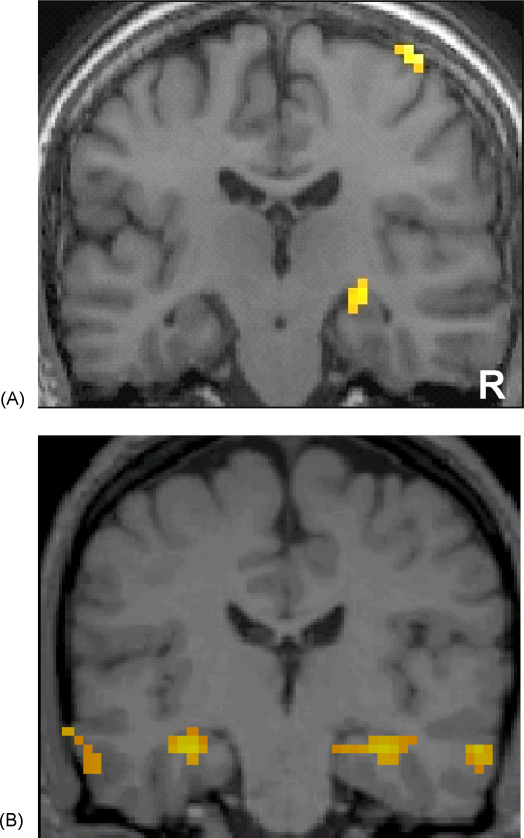
FMRI data from adult-acquired case P01 and developmental amnesia patient Jon. Both data sets were acquired on the same 1.5 T MRI scanner. (A) P01's right hippocampus was active during the incidental acquisition of facts (data from Hassabis, Kumaran, Vann, et al., 2007 – Supplementary Material). (B) Jon's hippocampi were active during an autobiographical memory recall task (data from Maguire et al., 2001).

Whilst the findings of Hassabis, Kumaran, Vann, et al. (2007) of deficient performance on the imagination task following hippocampal pathology are supportive of the concept of scene construction, it is notable that one of the five patients in that study was unimpaired. P01 (Hassabis, Kumaran, Vann, et al., 2007; also known as KN – Aggleton et al., 2005; McKenna & Gerhand, 2002), despite being profoundly amnesic for past experiences, was able to achieve rich scene construction with clear spatial coherence that was at the top end of the range of control participants. P01's pathology was acquired in adulthood, leaving him with almost 50% volume loss in both hippocampi (Aggleton et al., 2005). He was also very impaired on tests of recall, both for anterograde episodic memory and retrograde memory for autobiographical events, for which he had virtually no reliable recollections (Hassabis, Kumaran, Vann, et al., 2007). Despite this, his IQ was in the high average range, and he performed within normal limits on a number of tests of recognition (Aggleton et al., 2005). He also retained some ability to acquire new semantic information (McKenna & Gerhand, 2002). FMRI scanning revealed that there was residual BOLD activity in his right hippocampus during the incidental (and successful) acquisition of facts (Fig. 1a; see Hassabis, Kumaran, Vann, et al., 2007 – Supplementary Material; see also Maguire & Frith, 2004). Hassabis, Kumaran, Vann, et al. (2007) speculated that P01 may have retained a limited degree of residual hippocampal functionality that supported his performance on the imagination task.

P01 is just one patient who has preserved scene construction ability, and it is possible that he is atypical, making his relevance for understanding hippocampal function and the imagination of future scenarios uncertain. In order to understand more about the circumstances in which the ability to imagine fictitious and future scenarios might be preserved in the context of hippocampal damage, in the first instance it would be informative to identify other patients who perform similarly to P01 on such tasks. We noted that P01 has several features in common with another group of patients, namely those with developmental amnesia (DA). Patients with DA, a syndrome caused by relatively selective damage to the hippocampus following hypoxic-ischaemic episodes sustained in childhood, are able to acquire normal levels of intelligence and general knowledge despite a severe impairment in remembering the events of daily life (Vargha-Khadem et al., 1997). This dissociation between semantic and episodic memory (Tulving, 1972), seems to be accompanied by a second dissociation, between relatively preserved recognition ability and a marked impairment in recall (Adlam, Malloy, Mishkin, & Vargha-Khadem, 2009; Baddeley, Vargha-Khadem, & Mishkin, 2001; Mishkin, Suzuki, Gadian, & Vargha-Khadem, 1997; Vargha-Khadem et al., 1997). One particularly well-characterised patient with DA is Jon (see case summary below, and e.g. Adlam et al., 2009; Baddeley et al., 2001; Brandt, Gardiner, Vargha-Khadem, Baddeley, & Mishkin, 2008; de Haan, Mishkin, Baldeweg, & Vargha-Khadem, 2006; Gadian et al., 2000; Gardiner, Brandt, Vargha-Khadem, Baddeley, & Mishkin, 2006; Hartley et al., 2007; Mishkin et al., 1997; Vargha-Khadem et al., 1997). As well as the primary features of DA noted above, like P01 with adult-acquired pathology, Jon has ∼50% bilateral hippocampal volume loss in the face of a high average IQ. Interestingly, Jon appears to have preserved recollection of a small set of autobiographical events, that when recalled during fMRI were associated with bilateral activation of his residual hippocampal tissue (Maguire, Vargha-Khadem, & Mishkin, 2001; see Fig. 1b).

Given the similarity in profiles between developmental case Jon and adult-acquired case P01, we wondered how Jon would fare in constructing imagined scenarios. The commonalities between both cases led us to hypothesise that Jon too might be unimpaired. In order to examine this, we administered exactly the same tests of imagining fictitious and future scenarios to Jon as those undertaken by P01 and the other adult-acquired cases of hippocampal pathology described by Hassabis, Kumaran, Vann, et al. (2007).

## 2 Methods

### 2.1 Case description

Jon, who was 28 years old at time of testing, is a well-documented case of developmental amnesia (see above). Briefly, he was born prematurely at 26 weeks of gestation. He weighed less than 1 kg, suffered breathing problems and during his first 6 weeks of life required intubation and positive pressure ventilation for severe apnea (Gadian et al., 2000). He subsequently showed steady improvement and normal development, but by the age of five, memory problems were noted, and have since continued to be prominent. Direct measurement of Jon's MRI scans in adulthood indicated a reduction of ∼50% in the volume of both left and right hippocampal regions, with no evident pathology in the rest of the medial temporal lobe (Gadian et al., 2000; Vargha-Khadem et al., 1997). Consistent with his hippocampal abnormality, Jon has difficulty in reliably finding his way. He also tends to forget where belongings are normally kept, has problems remembering everyday events such as TV programmes just seen and is typically unable to give a detailed account of his activities earlier in the day.

On the other hand, he has a full scale IQ of 114 (high average), and performs normally on tests of reading, syntax, semantics and vocabulary (see Baddeley et al., 2001). He was able to attend normal school and acquire and retain the necessary semantic information that this involves. However, he performs poorly on a range of standardized memory tests, particularly when these involve recall rather than recognition. His performance on measures of recognition is relatively well preserved; he performs at a comparable level to control participants on a number of tests (Baddeley et al., 2001), or at a slightly lower level on others (Gardiner et al., 2006).

### 2.2 Other participants

Jon's performance was evaluated with respect to data from a previous study (Hassabis, Kumaran, Vann, et al., 2007), where exactly the same task and procedures were employed. In that study, five patients with amnesia took part (all male) each with primary damage to the hippocampi bilaterally that was acquired in adulthood. Full details of each patient are provided in Hassabis, Kumaran, Vann, et al. (2007). Briefly, the mean age of the patients was 52.8 years (SD 18.5, range 24–70), years of education 14.0 years (SD 3.7, range 11–19) and verbal IQ was 103.2 (SD 11.7, range 90–116). All of the patients had significant impairment of anterograde memory, some deficient on both recognition and recall tests, others on recall tests alone. Retrieval of pre-morbid semantic memory was intact in all cases, whilst retrograde memory for episodic experiences was impaired, with the amnesic period ranging from 10 years to a complete lifetime. Lesions were confirmed by structural MRI scans, and appeared to implicate the hippocampi, with no evidence of damage in adjacent medial temporal areas.

Besides the basic profile of hippocampal amnesia shared by Jon and the adult-acquired cases, P01 had additional features in common with Jon. As noted in the Introduction, P01, aged 46 at the time of testing, has been described in detail elsewhere (Aggleton et al., 2005; Hassabis, Kumaran, & Maguire, 2007; Hassabis, Kumaran, Vann, et al., 2007; McKenna & Gerhand, 2002). To summarise, this former industrial biochemist contracted meningeo-encephalitis at the age of 34 and then recurrent meningitis. He was left without useful motor function below T12, and amnesia. As with Jon, P01's IQ was in the high average range (113 at the time of this study, as measured by the WTAR, Wechsler, 2001); the volume loss in both his hippocampi was nearly 50%; and he also retained some ability to acquire new semantic information. P01 performed normally on tests of language, executive function, and perception. His anterograde memory for episodic information was grossly impaired. He performed within normal limits on some tests of recognition, but was very impaired on tests of recall. His retrograde memory for autobiographical events was grossly impaired across four decades.

Ten healthy control participants also took part (all male) in Hassabis, Kumaran, Vann, et al. (2007). The mean age of the control participants was 52.2 years (SD 16.9, range 25–76), years of education was 14.1 years (SD 2.8, range 11–17), and verbal IQ was 104.3 (SD 6.3, range 94–112). There was no significant difference between the patients with adult-acquired hippocampal damage and the control participants on these background characteristics (age *p* = 0.95; education *p* = 0.95; IQ *p* = 0.81). Each patient had two control participants who were matched to them on age and IQ. Of note, two of these control participants also matched Jon. All participants gave informed written consent to participation in the study in accordance with local research ethics committees.

### 2.3 Task and procedure

A full description of the task and scoring is provided in Hassabis, Kumaran, Vann, et al. (2007). Briefly, each participant was tested individually. The session was digitally recorded to enable transcription and later scoring of participants’ responses. The requirements of the task were explained, and several examples provided. During this practice phase we also established that patients could remember the instructions and the cues throughout a construction trial. Commonplace ordinary settings were chosen as scenarios to minimise the difficulty level, and be as independent from a participant's innate creative ability as possible. The scenarios purposely encompassed a variety of different subject matter from the man-made to the natural, and the busy to the isolated to ensure there were no content biases. Each participant completed 10 trials, 7 involving fictitious scenarios (a beach, museum, pub, port, market, forest, and castle setting). In three additional trials we also examined the effect of scenarios that were explicitly self-relevant and potentially plausible in the future (possible Christmas event, possible event over next weekend, possible future meeting with a friend).

For each scenario a short description was read out loud by the interviewer from a prepared script (e.g. ‘Imagine you’re lying on a white sandy beach in a beautiful tropical bay’) and the participant was instructed to vividly imagine the situation suggested by the cue and describe it in as much (multi-modal) detail as possible. Participants were explicitly told not to recount an actual memory or any part of one but rather create something new. A printed text card was placed on the desk in front of the participant summarising the main concept of the scenario to act as a reminder if needed. Participants were allowed to continue with their descriptions until they came to a natural end or they felt nothing else could be added. A probing protocol dictated the appropriate use of statements used by the examiner during the session. These mostly took the form of general probes encouraging further description (e.g. ‘can you see anything else in the scene?’), or asking for further elaboration on a theme introduced by the participant (e.g. ‘can you describe the fishing boat in more detail?’ in response to the participant saying ‘I can see a small fishing boat gently rocking out in the sea’). It was strictly prohibited for the examiner to introduce any concept, idea, detail or entity that had not already previously been mentioned by the participant. After each scenario, participants were asked to rate their constructions on a number of different parameters (see Section 2.4). At various points during a trial, and prior to the post-scenario ratings, the examiner verified that the participant still recalled the task instructions, the scenario in question, and the scenario he had created.

### 2.4 Scoring

A composite score, the *Experiential Index*, ranging from 0 to 60, measuring the overall richness of the imagined experience, was calculated from four sub-components.

*Content*: Each scenario description was segmented into a set of statements. Every statement was then classified as belonging to one of the four main categories: spatial reference, entity presence, sensory description, or thought/emotion/action. Repeated statements, irrelevant details and other tangential information that could not be classified into one of these four categories were discarded. Extensive pilot studies indicated that the production of seven details per category was an optimal reflection of performance whilst ensuring that those with more circuitous descriptions were not unfairly advantaged. Thus, the score for each details category was capped at a maximum of 7.

*Participant ratings*: Two subjective self-ratings contributed to the Experiential Index, each varying on a scale from 1 to 5: sense of presence (1 – ‘did not feel like I was there at all’; 5 – ‘felt strongly like I was really there’) and perceived salience (1 – ‘couldn’t really see anything’; 5 – ‘extremely salient’).

*Spatial Coherence Index*: As part of the feedback on each scenario participants were presented with a set of 12 statements each providing a possible qualitative description of the newly constructed experience. Participants were instructed to indicate the statements they felt accurately described their construction. They were free to identify as many or as few as they thought appropriate. Of the 12 statements, 8 were ‘integrated’ and indicated that aspects of the scene were contiguous (e.g. ‘I could see the whole scene in my mind's eye’) and 4 were ‘fragmented’ and indicated that aspects of the scene were not contiguous (e.g. ‘It was a collection of separate images’). One point was awarded for each integrated statement selected and one point taken away for each fragmented statement. This yielded a score between −4 and +8 that was then normalised around zero to give final Spatial Coherence Index score ranging between −6 (totally fragmented) and +6 (completely integrated). Any construction with a negative Spatial Coherence Index was considered to be incoherent and fragmented

*Quality judgement*: The final scoring component was the scorer's assessment of the overall quality of the construction. Scorers were instructed to rate how well they felt the description evoked a detailed ‘picture’ of the experience in their own mind's eye. Quality ratings could range from 0 (indicating the construction was completely devoid of details and with no sense of experiencing) to 10 (indicating an extremely rich and highly evocative construction that appeared to emerge from an extremely vivid imagining).

Several other ratings were also taken. After imagining each new experience, participants rated how difficult they found this on a scale from 1 to 5 (1 – very easy, …, 5 – very difficult). They were also asked to rate on a scale of 1 to 5 its similarity to an actual memory, in whole or in part (1 – nothing at all like any memories, …, 5 – exactly like a memory).

### 2.5 Data analysis

Our main interest was in comparing DA patient Jon with the group of 10 control participants. In order to do this we used a modified *t*-test (Crawford & Garthwaite, 2002; Crawford & Howell, 1998). This test treats an individual patient as a sample, affording the comparison of the patient and a reasonably small control group. All results are two-tailed with a significance threshold of *p* < 0.05.

## 3 Results

**Fig. 2:**
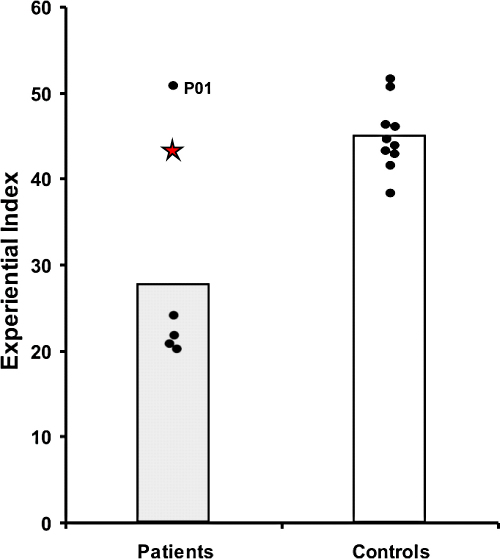
Scores on the Experiential Index. Data for each adult-acquired case of hippocampal damage and every control participant are represented by black dots (data from Hassabis, Kumaran, Vann, et al., 2007); the data point for P01 is noted. Vertical bars signify group means of the adult-acquired patients and the controls. The data point for developmental amnesia patient Jon is represented by a red star. (For interpretation of the references to colour in this figure legend, the reader is referred to the web version of this article.)

**Fig. 3:**
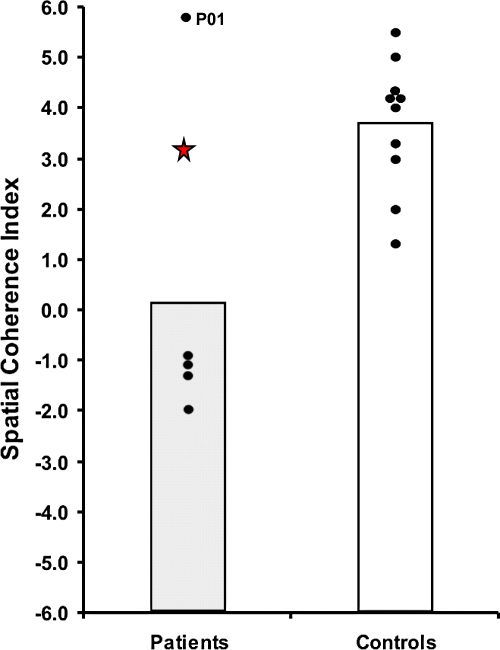
Scores on the Spatial Coherence Index. Data for each adult-acquired case of hippocampal damage and every control participant are represented by black dots (data from Hassabis, Kumaran, Vann, et al., 2007); the data point for P01 is noted. Vertical bars signify group means of the adult-acquired patients and the controls. The data point for developmental amnesia patient Jon is represented by a red star. (For interpretation of the references to colour in this figure legend, the reader is referred to the web version of this article.)

To provide a context for interpreting Jon's performance, we first provide a short summary of the findings from Hassabis, Kumaran, Vann, et al. (2007), where the group of 5 patients with adult-acquired hippocampal damage were compared to the 10 control participants (see Table 1, and Figs. 2 and 3). The patient group scored significantly lower on the overall Experiential Index than the control group, thus revealing that the ability to richly imagine new experiences is compromised in the context of bilateral hippocampal damage. Significant impairment was noted for the patients across all types of content (spatial references, entities present, sensory descriptions, thoughts/emotions/actions), and in the overall quality judgement. Interestingly, there was no difference between patients and controls in terms of perceived difficulty of the task, perceived sense of presence and perceived salience of the imagined scenarios, or in the degree of similarity to real memories. Of particular note were the scores on the Spatial Coherence Index, a measure of the contiguousness and spatial integrity of the imagined scene. Compared with controls, feedback from the patients indicated that their imagined experiences were fragmentary and lacking in coherence. Finally, whilst overall the patient group was impaired at imagining new experiences, examination of Figs. 2 and 3 shows that one of the patients (P01) was unimpaired on the task.

Jon's scores are also reported Table 1. Performances on the two scenario types (fictitious and personal plausible future events) were initially analysed separately. However, both had identical patterns of results, and so for clarity we present the results collapsed across scenarios. There was no significant difference between Jon and the control participants on the overall Experiential Index (*t*(9) −0.54, *p* = 0.61). Fig. 2 shows that his score was in the mid-range of the controls, and clearly significantly better than the patients with adult-acquired hippocampal damage (excluding P01). Similarly, he performed comparably to controls on measures of content–spatial references (*t*(9) 0.02, *p* = 0.99), entities present (*t*(9) −0.43, *p* = 0.68) and thoughts/emotions/actions (*t*(9) 0.92, *p* = 0.38). Interestingly, his score for sensory descriptions was borderline impaired (*t*(9) −2.172, *p* = 0.058), and this is the only content score on which Jon and the adult-acquired damage patients performed similarly (Jon's mean: 4.00, patient group mean: 4.12). Jon's overall quality judgement score was unimpaired (*t*(9) −0.13, *p* = 0.90). As with the patient group, there was no difference between Jon's ratings and those of control participants for sense of presence (*t*(9) 1.07, *p* = 0.31), perceived salience (*t*(9) 0.46, *p* = 0.67), task difficulty (*t*(9) −0.79, *p* = 0.45), and similarity to real memories (*t*(9) −0.05, *p* = 0.96). His score on the Spatial Coherence Index was also indistinguishable from controls (*t*(9) −0.43, *p* = 0.68, see Fig. 3).

**Fig. 4:**
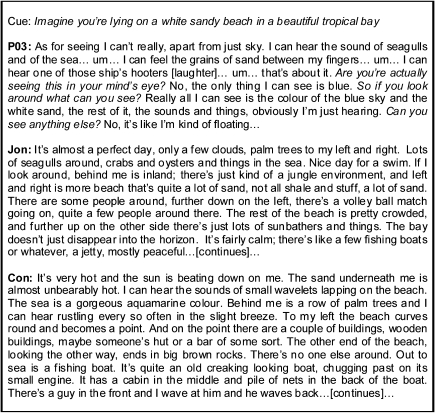
Examples of imagined scenarios. Representative excerpts from transcriptions relating to one of the scenarios, with the cue at the top. An excerpt from P03, an adult-acquired case of a similar age and IQ to Jon, is shown on top, followed by Jon's construction of the same scenario, followed by that of a control participant similar to both patients on age and IQ. Interviewer's probing comments are in italics. Relevant background information is noted in square brackets.

Considering the two control participants that were matched to Jon in terms of age and IQ, Experiential Index scores were similar (Jon's mean: 42.80, mean of the two controls: 47.83, SD 5.44), as was the Spatial Coherence Index (Jon's mean: 3.10, mean of the two controls: 3.75, SD 0.63). By contrast, a patient with adult-acquired hippocampal damage who was similar in age and IQ to Jon (P03), was severely impaired on the Experiential (20.30) and Spatial Coherence (−1.30) Indices, showing that merely having a high IQ is not sufficient to mitigate poor performance on the construction task. Fig. 4 depicts excerpts from P03, Jon and a matched control participant on one of the scenarios. Interestingly, Jon's performance on the construction task is broadly consistent with that of the adult-acquired case P01, who scored normally on all measures, including sensory details (P01's mean for this was 5.30).

After testing was complete, Jon was asked about his experience of doing the task. He observed: “I find it difficult to visualise things in my mind's eye. When I do try, I can do it. It doesn’t come automatically, though. I know it probably does with most people. It's not something I used to be able to do, but I’ve worked on it a lot over the years.” He notes that routinely, on a day-to-day basis, he generally does not create scenes, but only does so if there “is no other way to deal with the situation”. He does not instantly picture a scene, he has to work at it. “It doesn’t come at the snap of a finger, like it does with other people, I have a starting point and then fill in the details.” When asked if he was a ‘visual’ person, he laughed and said “definitely not; I’m the complete opposite of a visual person”. By contrast, when P01 was asked the same questions about how he was able to conjure up imaginary scenes and scenarios, he said “when asked to imagine a scene, it comes in one shot, in an instant, it's automatic, it comes very easily. It feels like a real space, and I can explore it, move around and all the detail is there for me to see”. He added that he can easily imagine events or experiences that other people describe to him, and regards himself as a very visual person.

## 4 Discussion

The aim of this study was to assess whether Jon, a patient with bilateral hippocampal damage and developmental amnesia, was able to imagine fictitious and future experiences. In line with our hypothesis, we found that this most well-characterised of DA patients, was indeed able to richly imagine both types of scenario in a comparable manner to control participants, and that his constructions were spatially coherent. This stands in marked contrast to four of the five patients with adult-acquired hippocampal damage tested by Hassabis, Kumaran, Vann, et al. (2007), who were very significantly impaired on the same imagination task. Notably, Jon's performance was similar to P01, the only adult-acquired case tested by Hassabis, Kumaran, Vann, et al. (2007) who was unimpaired on the task.

Jon's preserved scene construction ability shows that P01 was not an isolated and atypical case, and that some confluence of factors can arise that leads to preserved scene construction in the context of hippocampal amnesia. What might that be? Both Jon and P01 have high average IQ's, activation of their residual hippocampal tissue on fMRI despite similar hippocampal volume loss of ∼50% bilaterally, preserved performance on a number of recognition memory tests, and a similar pattern of performance on a weather prediction task (Kumaran et al., 2007). The relationship between these features, and possible causes and effects are difficult to determine. For instance, it may be that their semantic knowledge in some way boosts performance on scene construction tests. The relationship between semantic knowledge and the elements that comprise a constructed scenario/simulation remains to be determined, however (Hassabis & Maguire, 2009; Schacter & Addis, 2009; Summerfield et al., 2010; Szpunar, 2010). Moreover, the basis of Jon's (and the other DA cases) semantic knowledge acquisition is still essentially unknown.

It could also be the case that activity in their residual hippocampal tissue supports the ability to imagine new scenarios, and that this is the key feature. Residual hippocampal tissue was active in both patients (bilaterally in Jon, right hippocampus in P01) and in similar circumstances to control participants during autobiographical memory retrieval in Jon and semantic learning in P01. Whilst we cannot definitely relate function to these hippocampal activations, we suggest the activations might indicate some preserved hippocampal function which is also sufficient to support their preserved ability to imagine scenarios. Clearly, it will be important in the future to examine Jon and P01 and any similar cases that are reported, with fMRI whilst they construct imagined scenes or scenarios in order to verify that the hippocampus is indeed activated. Moreover, it will be of interest to know if the other brain areas that activate in control participants during imagination/simulation (Addis et al., 2007; Hassabis, Kumaran, & Maguire, 2007; Spreng et al., 2009) are also activated in such patients.

It is also important to bear in mind that just because P01 and Jon are both preserved at scene construction, does not imply that the underlying mechanisms are the same in both cases. The feedback provided by both patients during the debriefing session suggests they might have employed different strategies. P01's description of how he imagined fictitious scenarios resonates with that of control participants, in that it was automatic and a scene ‘appeared’ in an instant in his imagination. Jon, by contrast, had to work at visualising scenes, it was an effortful process. This may suggest a compensatory strategy that he actively engaged during the imagination task. Interestingly, the one sub-measure that was borderline impaired in Jon, and on which he scored comparably with the impaired adult-acquired cases, was sensory descriptions. This content category consisted of statements describing (in any modality) properties of an entity (e.g. ‘the chair I’m sitting on is made of wood’) as well as general weather and atmosphere descriptions (e.g. ‘it is very hot’ or ‘the room is very smoky’). His poorer performance on this measure may betray the effortful nature of his visualising, with the fine details eluding his compensatory strategy (Moscovitch et al., 2005; see also Bird, Vargha-Khadem, & Burgess, 2008; Kumaran et al., 2007). P01, by comparison, was similar to control participants on this measure.

Overall, these factors further underscore the need to perform fMRI with these and other patients whose scene construction abilities are similarly preserved as they may engage different brain areas or there may be altered inter-regional connectivity (Maguire et al., 2001) depending on their strategies. Furthermore, Jon is just one example of a patient with developmental amnesia, albeit the most comprehensively tested, and we can now add preserved ability to construct imagined scenarios to his neuropsychological profile. However, this may not apply to other DA cases (e.g. see Kwan, Carson, Addis, & Rosenbaum, in press) and it will be important to test more of these patients in order to establish if intact scene construction is a robust signature of the developmental amnesia syndrome.

## Figures and Tables

**Table 1:** Performance on the imagination task.

	Adult-acquired amnesia (*n* = 5)^a^	Mean (SD) Jon	Controls (*n* = 10)^a^
*Overall richness*			
Experiential Index	27.54 (13.12)	42.80	45.06 (4.02)

*Sub-components*			
Content			
Spatial references	2.38 (1.82)	5.30	5.28 (1.15)
Entities present	4.94 (1.26)	6.30	6.49 (0.42)
Sensory descriptions	4.12 (1.03)	4.00	5.64 (0.72)
Thoughts/emotions/actions	2.76 (1.77)	4.90	5.52 (0.64)

Participant ratings			
Sense of presence	3.46 (1.15)	4.20	3.65 (0.49)
Perceived salience	3.52 (1.19)	4.30	3.88 (0.48)

Spatial coherence			
Spatial Coherence Index	0.10 (3.21)	3.10	3.68 (1.30)

Scorer rating			
Quality judgement	3.88 (2.70)	7.00	7.13 (0.96)

*Other ratings*			
Task difficulty	2.20 (1.07)	1.60	2.13 (0.64)
Similarity to real memories	2.37 (0.90)	2.00	2.03 (0.62)

a Data from Hassabis, Kumaran, Vann, et al. (2007).
